# Ovulatory Shifts in Women’s Attractions to Primary Partners and Other Men: Further Evidence of the Importance of Primary Partner Sexual Attractiveness

**DOI:** 10.1371/journal.pone.0044456

**Published:** 2012-09-12

**Authors:** Christina M. Larson, Elizabeth G. Pillsworth, Martie G. Haselton

**Affiliations:** 1 Department of Psychology, University of California Los Angeles, Los Angeles, California, United States of America; 2 Department of Anthropology, University of California Los Angeles, Los Angeles, California, United States of America; 3 Department of Communication Studies, University of California Los Angeles, Los Angeles, California, United States of America; German Primate Centre, Germany

## Abstract

Previous research has documented shifts in women’s attractions to their romantic partner and to men other than their partner across the ovulation cycle, contingent on the degree to which her partner displays hypothesized indicators of high-fitness genes. The current study set out to replicate and extend this finding. Forty-one couples in which the woman was naturally cycling participated. Female partners reported their feelings of in-pair attraction and extra-pair attraction on two occasions, once on a low-fertility day of the cycle and once on a high-fertility day of the cycle just prior to ovulation. Ovulation was confirmed using luteinizing hormone tests. We collected two measures of male partner sexual attractiveness. First, the women in the study rated their partner’s sexual attractiveness. Second, we photographed the partners and had the photos independently rated for attractiveness. Shifts in women’s in-pair attractions across the cycle were significantly moderated by women’s ratings of partner sexual attractiveness, such that the less sexually attractive women rated their partner, the less in-pair attraction they reported at high fertility compared with low fertility (partial *r = *.37, *p*_dir_ = .01). Shifts in women’s extra-pair attractions across the cycle were significantly moderated by third-party ratings of partner attractiveness, such that the less attractive the partner was, the more extra-pair attraction women reported at high relative to low fertility (partial *r = *−.33, *p*_dir_ = .03). In line with previous findings, we found support for the hypothesis that the degree to which a woman’s romantic partner displays indicators of high-fitness genes affects women’s attractions to their own partner and other men at high fertility.

## Introduction

For humans, the window of fertility within the ovulation cycle lasts for just a few short days prior to ovulation [1]. Throughout human evolutionary history it was within this crucial window that women’s sexual decisions could have dramatic effects on fitness through conception and, ultimately, the birth of a child. As a result, women’s sexual decision-making is likely to have evolved to be sensitive to changes in fertility across the cycle. One specific hypothesis is that women’s attraction to men who display cues indicating that they possess genes that would have contributed to offspring viability or attractiveness in ancestral environments (i.e., *high-fitness genes*) are heightened on fertile days of the cycle [2]–[5]. In support of this hypothesis, a large number of studies document systematic shifts in women’s mate preferences across the ovulatory cycle. These studies demonstrate that, on high-fertility days of the cycle relative to low-fertility days, women express an increased preference for hypothesized indicators of high-fitness genes, including muscularity [6], masculine bodies [7], sexually dimorphic height [8], masculine facial features [9]–[10], masculine voices [11]–[12], socially dominant behavior [6] [13]–[14], and low levels of fluctuating asymmetry [15]–[18].

An implication of these preference shifts is that a woman’s attractions to her own primary romantic partner (*in-pair attraction*) and to men other than her partner (*extra-pair attraction*) might also vary across the ovulatory cycle, contingent upon the degree to which a woman’s partner displays cues of high-fitness genes. In ancestral environments, if women’s primary partner did not display cues of high-fitness genes, women could have enhanced their reproductive success by desiring and pursuing extra-pair affairs at high fertility, and possibly by *not* desiring or pursuing sex with their own partner at high fertility. If women’s primary partner did display cues of high-fitness genes, desiring and pursuing extra-pair affairs at high fertility probably did not enhance a woman’s reproductive success, but desiring and pursuing sex with her own partner at high fertility could have.

In addition to the reproductive benefits associated with choosing a partner with high-fitness genes, women may have benefitted historically from choosing a partner with genes compatible with her own. Genes in the Major Histocompatibility Complex (MHC) code for cell surface markers used to detect pathogens that have invaded a host’s body. MHC alleles are expressed co-dominantly (both paternally- and maternally-inherited alleles are expressed). Therefore, individuals who inherit different alleles from each parent have more complex cell surface markers than individuals whose parents share MHC alleles, improving their body’s ability to recognize and respond to a wide array of pathogens [19]. Evidence indicates that individuals prefer the body odors of potential partners with whom they share fewer MHC alleles [20]. Therefore, researchers hypothesize that genetic compatibility moderates cycle shifts in attraction in the same way that cues of high-fitness genes do.

A number of studies support these predictions (summarized in Table 1). The critical test in these studies is whether women’s fertility and her partner’s possession of cues of high-fitness genes interact to predict women’s in-pair and extra-pair attractions. Research consistently shows that when women’s partners are relatively low on indicators of high-fitness genes, women experience heightened *extra-pair attraction* at high relative to low fertility [21]–[25]. However, evidence for changes across the cycle in *in-pair attraction* is less consistent, with only two studies finding that women whose partners are relatively high on indicators of high-fitness genes experience heightened attraction to their own partners at high relative to low fertility [21]–[22].

**Table 1:** Moderating Effects of Cues of High-Fitness Genes on Shifts in Women’s Extra-Pair and In-Pair Attraction across the Ovulation Cycle.

Study	Moderator of Shiftsin Attraction	Source of Moderator	Extra-Pair Attraction	In-Pair Attraction
Haselton & Gangestad, 2006	Male Partner SexualAttractiveness	Female Partner Rating	Significant Interaction: Greater Upward Shift at Ovulation among Women with Less Attractive Partners	No Association
Pillsworth & Haselton, 2006	Male Partner SexualAttractiveness	Female Partner Rating	Significant Interaction: Greater Upward Shift at Ovulation among Women with Less Attractive Partners	No Association
Gangestad, Thornhill, & Garver-Apgar, 2005	Male Partner Fluctuating Asymmetry	Researcher Measurement	Significant Interaction: Greater Upward Shift at Ovulation among Women with Less Symmetrical Partners	Significant Interaction: Greater Upward Shift at Ovulation among Women with More Symmetrical Partners
Gangestad, Thornhill, & Garver-Apgar, 2010	Male Partner FacialMasculinity	Researcher Measurement	Significant Interaction: Greater Upward Shift at Ovulation among Women with Less Masculine Partners	No Association
Gangestad, Thornhill, & Garver-Apgar, 2010	Male Partner FacialAttractiveness	3^rd^ Party Rating of Photo	No Association	Significant Interaction: Greater Upward Shift at Ovulation among Women with More Attractive Partners
Garver-Apgar, Gangestad,Thornhill, Miller & Olp, 2006	Shared MHC Alleleswith Male Partner	Researcher Measurement	Significant Interaction:Greater Upward Shift atOvulation among Women whoShare fewer MHCAlleles with Partners	No Association
Current study	Male Partner SexualAttractiveness	Female Partner Rating	No Association	Significant Interaction: Greater Downward Shift at Ovulation among Women with Less Attractive Partners
Current study	Composite of Male Partner Body andFacial Attractiveness	3^rd^ Party Rating of Photo	Significant Interaction: Greater Upward Shift at Ovulation among Women with Less Attractive Partners	No Association

As can be seen in Table 1, researchers have investigated and found that several different traits hypothesized to indicate high-fitness genes moderate shifts in women’s attractions across the cycle. These include men’s fluctuating asymmetry [21], men’s facial masculinity [22], and the couple’s genetic compatibility [23]. If women who preferred mates displaying cues of high-fitness genes had higher reproductive success historically, women could have evolved to view men who possess cues of high-fitness genes as sexually attractive [6] [26]–[28]. Indeed, several studies have investigated and found that men’s sexual attractiveness [24]–[25] and facial attractiveness [22] moderate shifts in women’s attractions across the cycle. To examine whether men’s attractiveness moderates shifts in attraction, some studies used women’s ratings of their own partner [24]–[25], some have used researcher measurements of male partners [21]–[22], and one used third-party ratings of photographs of male partners [22].

The current study was designed to attempt to replicate and extend these findings. Unlike previous studies, this study included both women’s assessments of their own partner and third-party assessments of the partner based on a photograph. Body attractiveness is one feature that contributes to male sexual attractiveness [29], and preferences for attractive male bodies increase at high relative to low fertility [6]–[7]. However, body attractiveness has not previously been investigated as a moderator of ovulatory shifts in women’s in-pair and extra-pair attractions. In this study, we included ratings of men’s bodies as well as their faces, with the prediction that third-party assessments of body and facial attractiveness would moderate shifts in women’s attractions across the cycle in similar ways.

## Methods

### Ethics Statement

This study was approved by the UCLA Office of the Human Research Protection Program. All participants provided written informed consent.

### Participants

Participants were 41 heterosexual couples in which the female partner was naturally cycling (i.e., had not used any form of hormonal contraceptives within the past three months, was not pregnant or breastfeeding a child). Couples were ineligible if the woman reported that her average cycle length was less than 24 or more than 35 days long, or her rated confidence in her cycle length was less than 7 (1 = not at all confident; 9 = very confident) and she reported that she was usually off in her estimation of her next menstrual onset by more than four days. Couples were recruited from the UCLA campus and participated for payment or to fulfill course research requirements. The mean age of female participants was 21.0 years (*SD* = 4.3, range = 18–37); 43.9% self-identified as Asian, 17.1% as Hispanic, 12.2% as Caucasian, 2.4% as African American, and 24.3% as “other” or multiple ethnicities. The mean age of male participants was 22.0 years (*SD* = 4.5, range = 17–38); 41.5% self-identified as Asian, 17.1% as Hispanic, 17.1% as Caucasian, 4.9% as African American, and 19.4% as “other” or multiple ethnicities. Mean relationship length was 26.2 months (*SD* = 32.8, range = 1–192 months).

Thirty-two additional couples were originally recruited for the study but excluded from analyses because the female partner failed to complete all three sessions of the study (*n* = 19); showed no evidence of a luteinizing hormone (LH) surge, indicating impending ovulation (*n* = 3); or completed sessions outside of the predetermined high- or low-fertility windows (*n* = 10; see *Scheduling and LH testing* section). Of the couples who completed all parts of the study, 75.9% were eligible for inclusion in the analyses, a retention rate that is comparable to previous studies using similar inclusion criteria (e.g., 74.1% retention rate reported in one prior study [25], and 61.4% reported in another [30]).

### Scheduling and LH Testing

Female participants completed a total of three sessions: an initial session, a high-fertility session, and a low-fertility session. Male participants completed only the initial session. Following prior methods [30], high-fertility sessions were scheduled to occur between 16 and 19 days prior to a woman’s next predicted menstrual onset, and low-fertility sessions were scheduled to occur between 3 and 10 days prior to a woman’s next predicted menstrual onset (next menstrual onset was predicted based on the cycle information women provided at the initial session, described below). If a woman’s next predicted menstrual onset was between 4 and 17 days away, she was scheduled to complete her low-fertility session first (*n = *13); if not, she was scheduled to complete her high-fertility session first (*n = *28). Order of sessions (high or low first) is controlled for in the analyses below.

Beginning two days before their high-fertility session, women took a nationally marketed ovulation test (ClearBlue) daily for five days. These midstream urine tests document the surge in luteinizing hormone (LH) that occurs 24 to 48 hours prior to ovulation, and have been shown to be 97% concordant with ovulation confirmed by ultrasonography [31]. An LH surge was observed, on average, 0.7 days after the high-fertility session (*SD* = 1.5, range = −2–3).

Participants reported the date of menstrual onset between their low- and high-fertility sessions if they completed their low-fertility session first, and were asked to report the date of menstrual onset after their final session via postcard. If women did not report the date of menstrual onset, the date of predicted menstrual onset was used instead (for 32.9% of sessions). Based on these dates, high-fertility sessions occurred, on average, 16.8 days before menstrual onset (*SD* = 1.4, range = 14–20), and low-fertility sessions occurred, on average, 6.1 days before menstrual onset (*SD* = 2.5, range = 1–11). Ovulation typically occurs 14 to 15 days prior to menstrual onset, and unprotected sex is most likely to result in conception if it occurs on the six days leading up to and including ovulation [32]. Therefore, all women in our sample were considered to be in the high-fertility phase of the cycle during their high-fertility session, and none were considered to be in the high-fertility phase of the cycle during their low-fertility session.

### Initial Session

Both members of the couple completed their initial session at the same time, but in separate rooms. After providing their written informed consent, women provided information about their cycle regularity, cycle length, previous two dates of menstrual onset, and anticipated next menstrual onset, which was used to schedule their high- and low-fertility sessions.

Women next completed several computer-based questionnaires containing basic demographic items, partner ratings items, and control variable items. The partner rating items asked women to assess how sexually attractive they thought their romantic partner was to members of the opposite sex. Partner sexual attractiveness was the average of the following four questions, rated on a scale from 1 (*not at all*) to 9 (*extremely*): “How desirable do you think women find your partner as a short-term mate or casual sex partner, compared to most men,” “Compared to most men, how attractive is your partner’s body to women,” “Compared with most men, how attractive is your partner’s face to women,” and “How sexy would women say your partner is, compared to most men.” Women next completed several items assessing control variables. They reported their relationship length, completed the Sociosexuality Orientation Inventory (SOI) [33], reported whether or not they had engaged in sexual intercourse with their partner, and completed several relationship measures averaged into a relationship quality composite. The relationship quality composite was made up of the following four measures (α = .85): the Inclusion of Other in Self Scale [34], and the commitment, satisfaction, and relationship investment items from the Rusbult Investment Model Scale [35]. Women’s assessments of their relationship vary depending on their fertility status [36]. To account for this, residual relationship quality scores, controlling for fertility levels at the initial session (estimated using actuarial data [1]), were computed. These residual scores were used in place of raw scores in the analyses below.

After providing their written informed consent, men completed a computer-based questionnaire containing basic demographic items. Two digital photographs of the men were then taken: one full body and one close-up of the face. For both photographs, men were instructed to look straight into the camera and maintain a neutral expression. Eleven female research assistants (mean age 20.82) later rated the photographs. Photos were presented to the raters in a power-point presentation, one participant presented per slide with both the full body and the close-up of the face presented at the same time. Raters scrolled through the entire presentation, then went back to the beginning and rated how physically attractive each man’s body was and how physically attractive each man’s face was in comparison to other men at UCLA on a scale from 1 (*not at all*) to 9 (*extremely*). Ratings of body and facial attractiveness were averaged together to form one composite measure of third-party rated attractiveness. Four men did not consent to having their photograph taken, lowering the degrees of freedom in analyses using third-party ratings.

### High- and Low-fertility Sessions

At both their high- and low-fertility sessions, women completed a computer-based questionnaire assessing current feelings of attraction. Women rated how they felt over the past 48 hours, relative to other days, on a scale from −4 (*far less than usual*) to 4 (*far more than usual*). Five items assessed in-pair attraction: “Felt sexually attracted to partner,” “Thought that your partner looked physically attractive,” “Felt partner was higher than average on physical attractiveness,” “Wanted to have sex with your partner,” and “Felt receptive to physical attention by your partner.” Seven items assessed extra-pair attraction and flirtation: “Noticed attractive men around campus or around town,” “Flirted with someone you do not know,” “Flirted with acquaintances,” “Flirted with friends or co-workers,” “Been physically attracted to someone you do not know,” “Been physically attracted to an acquaintance,” and “Been physically attracted to a friend or co-worker.”

### Statistical Analyses

Repeated measures general linear models (SPSS 17.0) were used to analyze changes in women’s reports of in-pair and extra-pair attraction across the cycle. For all analyses, fertility status (high or low fertility) was a within-subjects repeated measure. Separate analyses were run for women’s reports of partner sexual attractiveness and composite third-party ratings of partner attractiveness. In follow-up analyses, third-party ratings of partner attractiveness were split into third-party ratings of body attractiveness and third-party ratings of facial attractiveness. In every case, the potential moderating variables were entered as quantitative covariates, and mean centered so the main effect of fertility would be estimated at the mean levels of these variables. When the interactions between fertility and the moderating variables were significant, simple effects analogs were run re-centering the moderating variables at one standard deviation below the mean, and one standard deviation above the mean. This allows for an estimation of the effects of fertility among women with low levels of partner attractiveness and among women with high levels of partner attractiveness. Order of sessions (high or low first) was entered as a between-subjects factor. Although analyses revealed some interactions with order, the main effect of fertility was not significant or marginally significant in any of these cases. Therefore, interactions with order are not discussed further.

Proximity to ovulation at the high-fertility session (as assessed by date of LH surge) was initially entered as a quantitative covariate to examine whether fertility effects were stronger among women who were closer to the day of ovulation at their high-fertility session. Proximity to menstrual onset at the low-fertility session was also initially entered as quantitative covariate to examine whether fertility effects were stronger among women who were closer to the day of menstruation at their low-fertility session, and therefore might be experiencing pre-menstrual symptoms. Neither of these variables significantly interacted with fertility in any of the analyses, therefore both variables were removed from the final models.

Table 2 presents overall descriptive statistics and the alpha reliabilities associated with women’s in-pair and extra-pair attraction ratings, women’s reports of partner sexual attractiveness, the composite measure of third-party rated attractiveness, and third-party ratings of body and face attractiveness, averaging across high and low fertility for women’s in-pair and extra-pair attraction ratings. Table 3 presents the correlations between the different moderators.

**Table 2:** Descriptive Statistics.

Variable	α	*M*	*SD*
Women’s ratings of partner sexual attractiveness	.86	3.52	1.23
Composite third-party ratings of partner body and face attractiveness	.90	3.15	0.93
Third-party ratings of partner body attractiveness	.84	3.40	0.94
Third-party ratings of partner facial attractiveness	.85	2.91	1.05
In-pair attraction	.79	0.95	0.93
Extra-pair attraction	.86	−0.51	0.96

*Note.* In-pair and extra-pair attraction are averaged across high and low fertility.

**Table 3:** Correlations Between Moderators.

Variable	1	2	3	4
1) Women’s ratings of partner sexual attractiveness	1.0	.46**	.45**	.41*
2) Composite third-party ratings of partner body and face attractiveness		1.0	.91***	.90***
3) Third-party ratings of partner body attractiveness	–		1.0	.71***
4) Third-party ratings of partner facial attractiveness	–		–	1.0

*Note.* *p<.05, **p<.01, ***p<.001.

In a second set of analyses, we added several control variables to examine whether the effects of interest were robust to the inclusion of variables that could be associated with women’s in-pair and extra-pair attractions. Whether or not a woman had had sex with her partner was entered as a between-subjects factor, and women’s logged relationship length, SOI score, and relationship quality composite score were added as quantitative covariates. One woman had an extremely high SOI score (six standard deviations above the mean). To reduce the influence of this outlier, this SOI score was brought down to the next highest SOI score. Relationship length (in months) was not normally distributed and was log transformed to normalize it. None of the interactions between fertility and any of the control variables were significant, but the results of all marginally significant interactions between fertility and control variables are reported.

Women differed from each other in fertility when they rated their partner’s sexual attractiveness at the initial session. To account for this, we estimated each woman’s fertility level at the initial session (using actuarial data, which provided the average probability that unprotected sex will result in conception on each day of the cycle [1]). Fertility levels at the initial session were not associated with women’s ratings of their partner’s sexual attractiveness (*r = *.09, *p* = .56). Furthermore, controlling for fertility levels at the initial session in analyses involving women’s assessments of their partner’s sexual attractiveness did not change the results reported below.

For analyses in which we had an a priori hypothesis about the direction of the effect, we used directed tests and report directed probabilities, as recommended by Rice and Gaines [37]. Directed tests allocate a probability of 0.04 (of a total α of 0.05) to the predicted direction and 0.01 to the unpredicted direction, thereby increasing the power to find anticipated effects, without eliminating the possibility of finding an effect in the unpredicted direction (in contrast to one-tailed tests which allocate a probability of 0.05 to one tail and 0 to the other tail). When results were in the predicted direction, the two-tailed *p* values obtained from SPSS were multiplied by 0.625, and when results were in the non-predicted direction, the two-tailed *p* values obtained from SPSS were multiplied by 2.5, and the standard cut-off value for significance was maintained at *p = *.05. When directed *p* values are reported, they are noted; all other analyses are traditional two-tailed tests.

The analyses also allowed for an investigation of between-subjects main effects of the partner attractiveness ratings on women’s extra-pair and in-pair attraction. None of the attractiveness ratings were significantly associated with women’s extra-pair attraction, averaged across high and low fertility. Women’s ratings of partner sexual attractiveness were marginally significantly associated with overall in-pair attraction, *F* (1, 38) = 3.92, *p = *.055; composite third-party ratings of attractiveness were marginally significantly associated with overall in-pair attraction, *F* (1, 34) = 3.68, *p = *.06; and third-party ratings of body attractiveness were significantly associated with overall in-pair attraction, *F* (1, 34) = 4.6, *p = *.04. In all cases, the more attractive the partner was, the more in-pair attraction women reported (sexual attractiveness *r = *.30, composite rated attractiveness *r = *.31, body attractiveness *r = *.34). Third-party ratings of facial attractiveness were not significantly associated with overall in-pair attraction.

## Results

### Women’s Ratings of Partner’s Sexual Attractiveness

#### In-pair attraction

**Figure 1:**
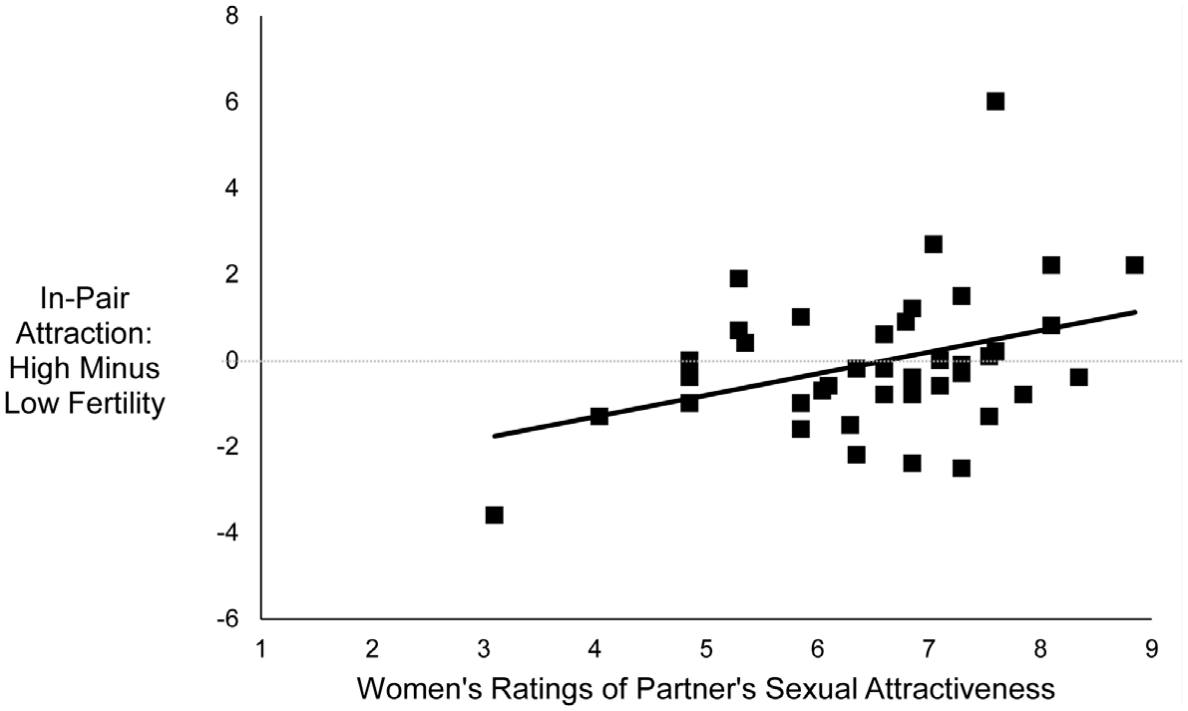
Relationship between women’s reports of in-pair attraction at high fertility, relative to low fertility, and women’s ratings of partner sexual attractiveness. Points represent residual scores controlling for order of sessions. *N* = 41, partial *r = *.37, *p*_dir_ = .01.

There was no main effect of fertility on women’s feelings of in-pair attraction, *F* (1, 38) = 0.34, *p*_dir_ = .35, partial η^2^ = .01. However, the interaction between fertility and women’s ratings of partner sexual attractiveness was significant, *F* (1, 38) = 6.05, *p*_dir_ = .01, partial η^2^ = .14. As shown in Figure 1, the less sexually attractive women rated their partner, the less in-pair attraction they reported at high fertility compared with low fertility (partial *r = *.37, *p*_dir_ = .01). Follow-up analyses revealed that when ratings of partner sexual attractiveness were one standard deviation below the mean, women reported significantly less in-pair attraction at high fertility than at low fertility, *F* (1, 38) = 4.75, *p*_dir_ *= *.02, partial η^2^ = .11 (marginal mean at high fertility = 0.30, *SD = *0.22; marginal mean at low fertility = 1.06, *SD = *0.31). However, when ratings of partner sexual attractiveness were one standard deviation above the mean, women’s reported in-pair attraction did not differ significantly between high and low fertility, *F* (1, 38) = 1.54, *p = *.22, partial η^2^ = .04. When the control variables were added to the analysis, the main effect of fertility remained non-significant, *F *(1, 34) = 0.03, *p*_dir_ = .54, and the interaction between fertility and partner sexual attractiveness remained significant, *F* (1, 34) = 3.63, *p*_dir_ = .04.

#### Extra-pair attraction

There was no main effect of fertility on women’s feelings of extra-pair attraction, *F* (1, 38) = 1.42, *p*_dir_ = .15, partial η^2^ = .04, and the interaction between fertility and women’s ratings of partner sexual attractiveness was also non-significant, *F* (1, 38) = 1.68, *p*_dir_ = .51, partial η^2^ = .04. When the control variables were added to the analysis, the main effect of fertility remained non-significant, *F* (1, 34) = 0.002, *p*_dir_ = .60, and the interaction between fertility and partner sexual attractiveness remained non-significant, *F* (1, 34) = 1.74, *p*_dir_ = .12.

### Composite Third-party Ratings of Partner’s Attractiveness

#### In-pair attraction

There was no main effect of fertility on women’s feelings of in-pair attraction, *F* (1, 34) = 1.08, *p*_dir_ = .19, partial η^2^ = .03, and the interaction between fertility and composite third-party ratings of partner attractiveness was also non-significant, *F* (1, 34) = 0.01, *p*_dir_ = .58, partial η^2^<.01. When the control variables were added to the analysis, the main effect of fertility remained non-significant, *F* (1, 30) = 0.19, *p*_dir_ = .41, and the interaction between fertility and rated attractiveness remained non-significant, *F* (1, 30) = 0.34, *p*_dir_ = .35.

The interaction between fertility and whether or not a woman had had sex with her partner was marginally significant, *F* (1, 30) = 3.46, *p* = .073. Ratings of in-pair attraction did not significantly differ between high and low fertility for either group of women (women who had not had sex with their partner: *F* (1, 5) = 0.001, *p = *.97; women who had had sex with their partner: *F* (1, 20) = 0.42, *p = *.53), but ratings of in-pair attraction were lower at high relative to low fertility more so among women who had not had sex with their partner than among women who had.

#### Extra-pair attraction

**Figure 2:**
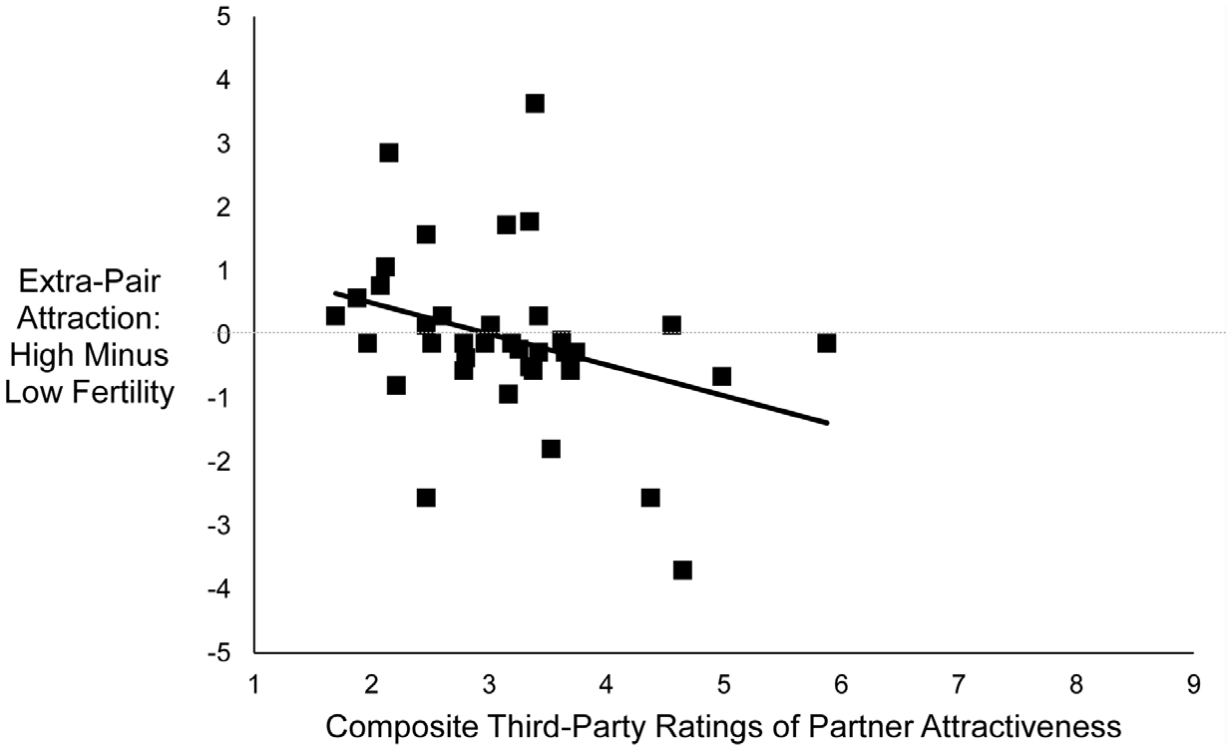
Relationship between women’s reports of extra-pair attraction at high fertility, relative to low fertility, and composite third-party ratings of women’s partner’s attractiveness. Points represent residual scores controlling for order of sessions. *N* = 37, partial *r = *−.33, *p*_dir_ = .03.

There was no main effect of fertility on women’s feelings of extra-pair attraction, *F* (1, 34) = 0.39, *p*_dir_ = .34, partial η^2^ = .01. However, the interaction between fertility and composite third-party ratings of partner attractiveness was significant, *F* (1, 34) = 4.16, *p*_dir_ = .03, partial η^2^ = .11. As shown in Figure 2, the less attractive a woman’s partner was rated, the more extra-pair attraction women reported at high fertility compared with low fertility (partial *r = *−.33, *p*_dir_ = .03). Follow-up analyses revealed that when women’s partners were one standard deviation below the mean on rated attractiveness, women reported significantly more extra-pair attraction at high fertility than at low fertility, *F* (1, 34) = 3.74, *p*_dir_ *= *.04, partial η^2^ = .099 (marginal mean at high fertility = −0.34, *SD = *0.29; marginal mean at low fertility = −0.94, *SD = *0.28). However, when women’s partners were one standard deviation above the mean on rated attractiveness, women’s reported extra-pair attraction did not differ significantly between high and low fertility, *F* (1, 34) = 0.88, *p = *.36, partial η^2^ = .02. When the control variables were added to the analysis, the main effect of fertility remained non-significant, *F* (1, 30) = 1.4, *p*_dir_ = .16, and the interaction between fertility and rated attractiveness remained significant, *F* (1, 30) = 4.73, *p*_dir_ = .02. The interaction between fertility and SOI was marginally significant, *F* (1, 30) = 3.02, *p* = .093, such that the more sociosexually restricted a woman was, the more extra-pair attraction she reported at high fertility compared with low fertility (partial *r = *−.30, *p* = .093).

### Third-party Ratings of Partner’s Body Attractiveness

#### In-pair attraction

There was no main effect of fertility on women’s feelings of in-pair attraction, *F* (1, 34) = 1.08, *p*_dir_ = .19, partial η^2^ = .03, and the interaction between fertility and third-party ratings of partner body attractiveness was also non-significant, *F* (1, 34) = 0.02, *p*_dir_ = .55, partial η^2^<.01. When the control variables were added to the analysis, the main effect of fertility remained non-significant, *F* (1, 30) = 0.2, *p*_dir_ = .41, and the interaction between fertility and rated body attractiveness remained non-significant, *F* (1, 30) = 0.12, *p*_dir_ = .46.

The interaction between fertility and whether or not a woman had had sex with her partner was marginally significant, *F* (1, 30) = 3.21, *p* = .083. Ratings of in-pair attraction did not significantly differ between high and low fertility for either group of women (women who had not had sex with their partner: *F* (1, 5) = 0.02, *p = *.88; women who had had sex with their partner: *F* (1, 20) = 0.37, *p = *.55), but ratings of in-pair attraction were lower at high relative to low fertility more so among women who had not had sex with their partner than among women who had.

#### Extra-pair attraction

**Figure 3:**
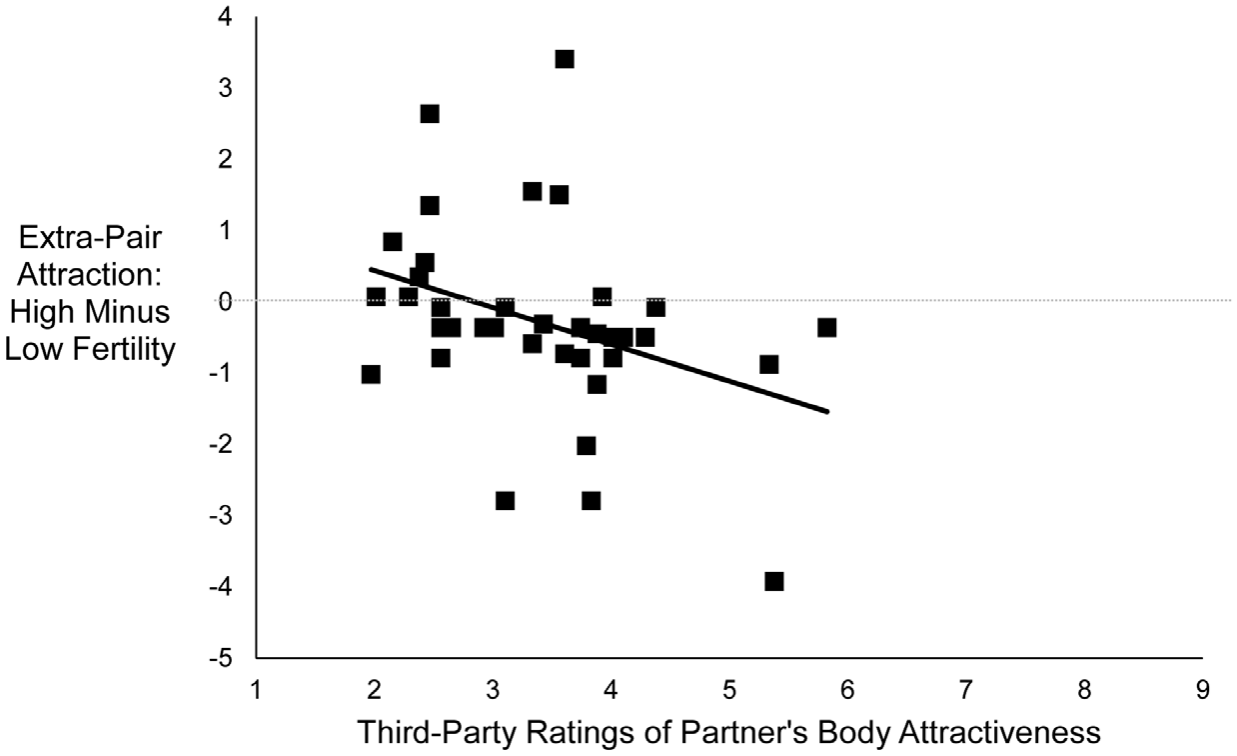
Relationship between women’s reports of extra-pair attraction at high fertility, relative to low fertility, and third-party ratings of women’s partner’s body attractiveness. Points represent residual scores controlling for order of sessions. *N* = 37, partial *r = *−.36, *p*_dir_ = .02.

There was no main effect of fertility on women’s feelings of extra-pair attraction, *F* (1, 34) = 0.47, *p*_dir_ = .31, partial η^2^ = .01. However, the interaction between fertility and third-party ratings of partner body attractiveness was significant, *F* (1, 34) = 5.08, *p*_dir_ = .02, partial η^2^ = .13. As shown in Figure 3, the less attractive a woman’s partner’s body was rated, the more extra-pair attraction women reported at high fertility compared with low fertility (partial *r = *−.36, *p*_dir_ = .02). Follow-up analyses revealed that when women’s partners were one standard deviation below the mean on rated body attractiveness, women reported significantly more extra-pair attraction at high fertility than at low fertility, *F* (1, 34) = 4.38, *p*_dir_ *= *.03, partial η^2^ = .11 (marginal mean at high fertility = −0.2, *SD = *0.29; marginal mean at low fertility = −0.85, *SD = *0.28). However, when women’s partners were one standard deviation above the mean on rated body attractiveness, women’s reported extra-pair attraction did not differ significantly between high and low fertility, *F* (1, 34) = 1.08, *p = *.31, partial η^2^ = .03. When the control variables were added to the analysis, the main effect of fertility remained non-significant, *F* (1, 30) = 1.8, *p*_dir_ = .12, and the interaction between fertility and rated body attractiveness remained significant, *F* (1, 30) = 5.34, *p*_dir_ = .02.

### Third-party Ratings of Partner’s Facial Attractiveness

#### In-pair Attraction

There was no main effect of fertility on women’s feelings of in-pair attraction, *F* (1, 34) = 1.08, *p*_dir_ = .19, partial η^2^ = .03, and the interaction between third-party ratings of partner facial attractiveness and fertility was also non-significant, *F* (1, 34) = 0.002, *p*_dir_ = .6, partial η^2^<.01. When the control variables were added to the analysis, the main effect of fertility remained non-significant, *F* (1, 30) = 0. 15, *p*_dir_ = .44, and the interaction between fertility and rated facial attractiveness remained non-significant, *F* (1, 30) = 0.54, *p*_dir_ = .29.

The interaction between fertility and whether or not a woman had had sex with her partner was marginally significant, *F* (1, 30) = 3.68, *p* = .065. Ratings of in-pair attraction again did not significantly differ between high and low fertility for either group of women (women who had not had sex with their partner: *F* (1, 5) = 0.12, *p = *.75; women who had had sex with their partner: *F* (1, 20) = 0.42, *p = *.52), but ratings of in-pair attraction were again lower at high relative to low fertility more so among women who had not had sex with their partner than among women who had.

#### Extra-pair attraction

**Figure 4:**
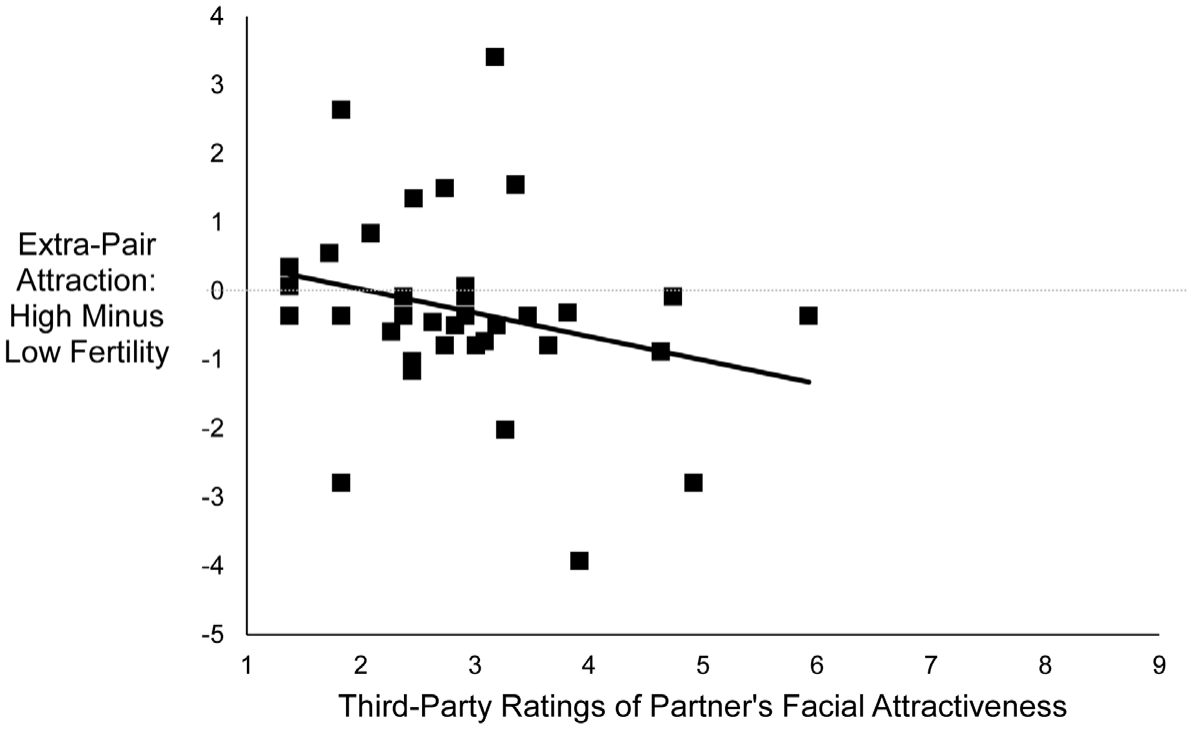
Relationship between women’s reports of extra-pair attraction at high fertility, relative to low fertility, and third-party ratings of women’s partner’s facial attractiveness. Points represent residual scores controlling for order of sessions. *N* = 37, partial *r = *−.26, *p*_dir_ = .08.

There was no main effect of fertility on women’s feelings of extra-pair attraction, *F* (1, 34) = 0.37, *p*_dir_ = .34, partial η^2^ = .01. However, the interaction between fertility and third-party ratings of partner facial attractiveness was marginally significant, *F* (1, 34) = 2.46, *p*_dir_ = .08, partial η^2^ = .07. As shown in Figure 4, the less attractive a woman’s partner’s face was rated, the more extra-pair attraction women reported at high fertility compared with low fertility (partial *r = *−.26, *p*_dir_ = .08). Follow-up analyses revealed that when women’s partners were one standard deviation below the mean on rated facial attractiveness, women reported marginally significantly more extra-pair attraction at high fertility than at low fertility, *F* (1, 34) = 2.57, *p*_dir_ *= *.07, partial η^2^ = .07 (marginal mean at high fertility = −0.48, *SD = *0.29; marginal mean at low fertility = −0.99, *SD = *0.27). However, when women’s partners were one standard deviation above the mean on rated facial attractiveness, women’s reported extra-pair attraction did not differ significantly between high and low fertility, *F* (1, 34) = 0.4, *p = *.53, partial η^2^ = .01. When the control variables were added to the analysis, the main effect of fertility remained non-significant, *F* (1, 30) = .98, *p*_dir_ = .21, and the interaction between fertility and rated facial attractiveness remained marginally significant, *F* (1, 30) = 3.05, *p*_dir_ = .06. The interaction between fertility and SOI was marginally significant, *F* (1, 30) = 2.89, *p* = .099, such that the more sociosexually restricted a woman was, the more extra-pair attraction she reported at high fertility compared with low fertility (partial *r = *−.30, *p* = .099).

## Discussion

In line with previous research, this study found support for the hypothesis that the degree to which a woman’s romantic partner displays cues of high-fitness genes affects her sexual attractions at high fertility within the ovulation cycle. The current study used both women’s own ratings of their partner’s sexual attractiveness and third-party ratings of partner attractiveness to test this hypothesis. Women’s ratings of their partner’s sexual attractiveness interacted with fertility to predict women’s in-pair sexual attractions, such that only women who rated their partners as relatively low on sexual desirability experienced decreases in in-pair attraction at high relative to low fertility. Women’s ratings of their partner’s sexual attractiveness did not interact with fertility to predict women’s extra-pair attractions. Third-party ratings of partner’s attractiveness interacted with fertility to predict women’s extra-pair attractions, such that only women whose partners were rated as relatively low on attractiveness experienced increases in extra-pair attraction at high relative to low fertility. Third-party ratings of men’s attractiveness did not interact with fertility to predict women’s in-pair attractions. Separate analyses revealed that third-party ratings of men’s body attractiveness significantly predicted fertility shifts in women’s feelings of extra-pair attraction, whereas third-party ratings of men’s facial attractiveness predicted these fertility shifts at only a marginally significant level.

As noted above, the results of this study broadly conform to those found in previous research. Specifically, when a woman’s male partner displays lower levels of cues hypothesized to indicate high-fitness genes, she is more likely to experience changes in her attractions across the cycle that are consistent with the hypothesis that women evolved to pursue high-fitness genes when most fertile within the cycle. Five previous studies found that the extent to which a woman’s male partner displayed hypothesized cues of high-fitness genes interacted with the woman’s fertility status to predict her feelings of extra-pair attraction. We also found this pattern, but only when we examined third-party ratings of male partner attractiveness as moderators of cycle shifts in attraction. Two previous studies found that the extent to which a woman’s male partner displayed hypothesized cues of high-fitness genes interacted with the woman’s fertility status to predict her feelings of in-pair attraction. We also found this pattern, but only when we examined women’s assessments of their partner’s sexual attractiveness as the moderator of cycle shifts in attraction.

This study provides the first demonstration within the same study that both third-party assessments of a man’s qualities and assessments made by his partner predict systematic cycle shifts in women’s attractions. This indicates that third parties can objectively observe the cues of high-fitness genes that predict women’s cycle shifts. It is noteworthy that in this study women’s own assessments of their partner’s sexual attractiveness were more strongly related to cycle shifts in in-pair attraction, whereas third-party assessments were more strongly related to cycle shifts in extra-pair attraction. In previous studies, women’s own assessments of their partners’ sexual attractiveness were more strongly related to cycle shifts in women’s extra-pair attraction [24]–[25], and third-party ratings were more strongly related to women’s cycle shifts in-pair attraction [22].

It is unclear why in some studies variables related to male partner attractiveness moderate shifts in women’s in-pair attraction, whereas in other studies these variables moderate shifts in women’s extra-pair attraction. In general, studies have been more likely to find cycle shifts in extra-pair attraction than in-pair attraction, but the underlying pattern is similar: When women’s partners display lower levels of cues of high-fitness genes, women are more likely to experience sexual attractions in line with a reproductive strategy of reduced attraction to the partner and/or heightened attraction to other men at high fertility.

This study used a rigorous methodology to assess cycle position, including the use of hormone tests. However, it also had limitations. First, participants were primarily undergraduates involved in dating relationships, limiting our ability to generalize beyond this population. Because our study and others like it use samples comprised primarily of young, nulliparous women who are in relatively new relationships, it remains unknown how the attraction dynamics we have found might play out in older women in more established relationships, including relationships that have produced children. Because women with children could have relied on childcare assistance from their male long-term partner, it is plausible that the costs of extra-pair mating, including abandonment by male partners upon the discovery of infidelity, were greater for ancestral women with children than for those without children. It is plausible that these costs often exceeded any benefit of extra-pair mating for high-fitness genes. If this was the case, the increases in extra-pair attraction at high fertility we have documented could be attenuated in women in more established long-term relationships, and particularly in long-term relationships that have produced children [38]. This should be examined in future research.

Second, because of the global nature of third-party ratings and women’s ratings of their partner, we were not able to examine the specific features in men that best predict shifts in women’s attractions (but see [6] [22]). Related to this point, we did not give male partners standardized clothing or make an effort to conceal clothing or hairstyle cues in the photos. These cues could also influence ratings and could be taken into account in future research.

Finally, because women provided responses at only two time points, we could not address the questions of whether changes in women’s attractions translate into changes in women’s behaviors over broader expanses of time, and what impact these changes have on women’s relationships. This limitation also indicates a fruitful avenue for future research.

Although the precise nature of cycle shifts varies across studies, past studies and the current study, taken together, strongly support the notion that there are shifts in women’s attraction across the ovulation cycle that are related to male primary partner attractiveness. This conclusion is important and noteworthy because it demonstrates a basic link between women’s hormone cycles and their sexual attractions, points to the underappreciated role of male sexual attractiveness in psychological research on attraction, and offers insight into a source of variation in women’s day to day relationship functioning.
